# Characteristics and outcomes in severe and critically ill children with first wave SARS-CoV-2 Omicron infection in Northeast China

**DOI:** 10.3389/fcimb.2025.1495783

**Published:** 2025-04-15

**Authors:** Tingting Sun, Yunhan He, Zeyu Wang, Lijie Wang, Chunfeng Liu, Wei Xu, Kai You

**Affiliations:** Department of Pediatrics, Shengjing Hospital of China Medical University, Shenyang, China

**Keywords:** COVID-19, Omicron variant, PICU, respiratory failure, neurological disorder

## Abstract

**Aim:**

To describe the characteristics of severe and critically ill children with first-wave SARS-CoV-2 Omicron infection admitted to the pediatric intensive care unit (PICU) at the National Children’s Regional Medical Center in Northeast China and to explore factors associated with poor outcomes.

**Methods:**

This observational cohort study was conducted in a PICU in northeastern China and included children under 18 years of age who were severely and critically ill due to SARS-CoV-2 Omicron infection between December 2022 and February 2023. Patients were categorized into two groups: the invasive mechanical ventilation (IMV) group and the non-IMV group. The primary outcome measured was the need for IMV, while secondary outcomes included death or prolonged PICU stay. Univariate and multivariate logistic regression analyses were performed to identify risk factors for poor outcomes.

**Results:**

A total of 38 severe and critically ill children were included in the study. Of these, 25 (66%) were diagnosed with respiratory failure, and four (16%) developed acute respiratory distress syndrome. Additionally, 21 (55%) were diagnosed with COVID-19-associated neurological disorders, and 18 (47%) received IMV. Multivariate logistic regression analysis identified the chest computed tomography (CT) score, based on the COVID-19 Risk Assessment and Diagnosis System (CO-RADS), was statistically significant as an independent predictor for IMV in severe and critically ill children (odds ratio [OR]: 2.781 [95% confidence interval (CI): 1.021–7.571]). Furthermore, the Pediatric Logistic Organ Dysfunction-2 (PELOD-2) score and serum aspartate aminotransferase (AST) levels at admission were found to be independent predictors of death or prolonged PICU stay.

**Conclusions:**

Respiratory failure and COVID-19-associated neurological disorders were the most common complications among severe and critically ill children with first-wave SARS-CoV-2 Omicron infection. Chest CT score, PELOD-2 score, and serum AST levels may serve as important indicators of poor outcomes in this patient population.

## Graphical Abstract

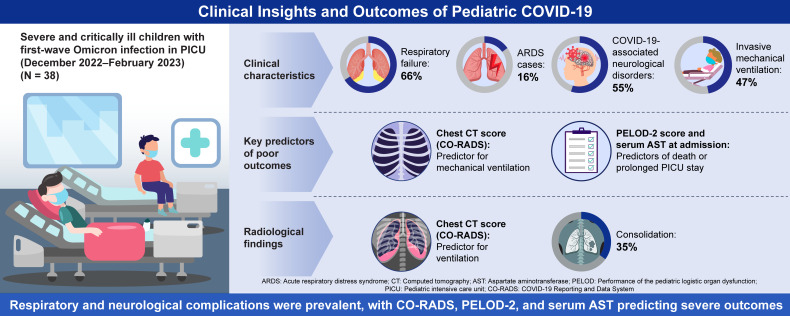

## 1 Introduction

Severe acute respiratory syndrome coronavirus type 2 (SARS-CoV-2), the causative agent of coronavirus disease 2019 (COVID-19), has spread globally since its emergence in late 2019 (Baker et al., 2022). As the virus continues to mutate, different COVID-19 variants have emerged with varying characteristics. The Omicron variant (B.1.1.529), first identified in South Africa in November 2021, became the predominant strain during the fourth wave of the global COVID-19 pandemic (World Health Organization, 2021). The Chinese government implemented strict epidemic control measures, including the “zero COVID” policy, which remained in effect until December 2022. After the zero-tolerance policy on COVID-19 ended in 2022, China experienced a widespread COVID-19 epidemic driven by the Omicron variant (Chinese Center for Disease Control and Prevention, 2023). Between December 2022 and February 2023, the cumulative number of confirmed cases increased by approximately 89.4 million, bringing the cumulative total to 99 million in China (World Health Organization, 2023a). During this period, all locally transmitted SARS-CoV-2 cases were confirmed to be caused by the Omicron variant according to the Chinese Center for Disease Control and Prevention.

Studies have shown that while the Omicron variant is more transmissible than previous variants, it is generally associated with lower disease severity and a reduced incidence of multisystem inflammatory syndrome in children (MIS-C) (Tian et al., 2022; Wang et al., 2022; Whittaker et al., 2022; Wolter et al., 2022; Choi et al., 2023; Ross et al., 2023; Wang et al., 2023). However, extra-pulmonary organ involvement, the need for invasive mechanical ventilation (IMV), and mortality rates in severe and critical cases have not significantly decreased (Corriero et al., 2022; de Prost et al., 2022). The high transmissibility of Omicron led to an increased medical burden on both adult and pediatric healthcare systems. Studies have reported a rapid rise in pediatric SARS-CoV-2 infections and hospitalizations during the first Omicron wave (Cloete et al., 2022; Menni et al., 2022). Although most pediatric cases are mild to moderate, some children develop severe illness requiring intensive care unit (ICU) admission (Bhalala et al., 2022; Uka et al., 2022; Zhang et al., 2023). There is limited research on children who have experienced severe or critical illness due to first-time infection with the Omicron variant.

In this study, we investigated children with severe and critical COVID-19 who were admitted to the pediatric ICU (PICU) of the National Regional Medical Center in Northeast China during the first Omicron epidemic and explored factors associated with poor outcomes.

## 2 Methods

### 2.1 Study design and population

In this retrospective study, we investigated children aged <18 years who were admitted to the largest PICU at Shengjing Hospital of China Medical University, which serves as the National Children’s Regional Medical Center in Northeast China. The study was approved by the institutional ethics committee (Ethics No. 2023PS30K). Medical records from the electronic medical record system were reviewed for patients admitted to the PICU between December 1, 2022, and February 28, 2023. All enrolled children had laboratory-confirmed COVID-19 infection, confirmed by real-time reverse-transcriptase polymerase chain reaction of nasopharyngeal swab samples. The Chinese Center for Disease Control and Prevention tested hospitalized patients to determine the COVID-19 strain during the epidemic, confirming that all cases were caused by the Omicron variant.

The inclusion criteria were: (a) Compliance with the diagnostic criteria for severe and critical cases as defined by the National Health Commission of the People’s Republic Of China (NHCC) (Sun et al., 2025) (b) Availability of clinical data.

The exclusion criteria were (a) Refusal to participate (b) Discontinuation of therapy, including self-discharge against medical advice.

### 2.2 Criteria for diagnosis and definition

According to the guidelines on the diagnosis and treatment of new coronavirus pneumonia (version 10), issued by the NHCC on January 5, 2023 (Sun et al., 2025), all cases were classified into four severity groups: mild, moderate, severe, and critical. Severe cases were defined as meeting at least one of the following conditions: Persistent high fever > 3 days, shortness of breath, SpO2 ≤ 93% on room air at rest, alar flapping, the presence of three concave signs or wheezing, unconsciousness, convulsions, or difficulty feeding with signs of dehydration. Critical cases were defined as meeting at least one of the following criteria: Respiratory failure requiring mechanical ventilation, shock, and ICU admission for other organ dysfunctions. Details of the diagnostic criteria and differences from the WHO guidelines (World Health Organization, 2023b) are provided in **Supplementary Table 1**. Only severe and critical cases were included in this study.

Data extracted from medical records included: Pediatric Critical Illness Score (PCIS) at admission (Zhang et al., 2025), Pediatric Logistic Organ Dysfunction (PELOD-2) score at admission (Leteurtre et al., 2013), and modified Rankin Scale (mRS) score at discharge (Quinn et al., 2009). The diagnostic criteria for pediatric acute respiratory distress syndrome (ARDS) were based on the guidelines established by the Second Pediatric Acute Lung Injury Consensus Conference (PALICC-2) group in 2023 (Emeriaud et al., 2023). The chest CT score was based on the COVID-19 Risk Assessment and Diagnosis System (CO-RADS) (Prokop et al., 2020), which was employed to evaluate the severity of lung involvement in COVID-19 pneumonia. Furthermore, we assessed the mRS score for several patients 90 days post-discharge.

### 2.3 Data collection and classification

Age, sex, preexisting medical conditions, clinical data (including symptoms, time of onset, clinical diagnosis, and presence of complications), laboratory data, radiological findings (X-ray, computed tomography [CT], and magnetic resonance imaging [MRI] characteristics), treatment strategies, and patient outcomes were collected. Data were verified directly from the electronic medical records by at least two members of the research group to reduce information and transcription biases.

Patients were categorized into two groups based on the use of IMV. A comprehensive analysis was conducted on demographic information, clinical characteristics, laboratory and imaging results, treatment, outcomes, and risk factors associated with poor outcomes in both groups. Primary outcomes were defined as the need for IMV, while secondary outcomes included death or prolonged PICU stay (defined as ≥ 14 days).

### 2.4 Statistical analysis

The data in this study did not follow a normal distribution. Therefore, quantitative data were presented as the median and interquartile range (IQR), while categorical variables were reported as numbers (percentages). Cases in which corresponding tests or examinations were not performed were excluded, and the results were expressed as the number of positive outcomes divided by the total number tested. The Wilcoxon signed-rank test was used to compare continuous variables, while the corrected chi-square test or Fisher’s exact test was applied for categorical variables. Bivariate regression analyses were conducted to screen variables for inclusion in multivariate regression models, which aimed to identify factors associated with the need for IMV and factors influencing prolonged PICU stay or death. Backward stepwise regression was applied in the multivariable analysis. Statistical significance was set at *p* < 0.05. Variables included in the final multivariate model were reported with odds ratios (ORs) and their 95% confidence intervals (CIs). All statistical analyses were performed using IBM SPSS Statistics version 25.0 (IBM Corp.).

## 3 Results

### 3.1 Demographic data and clinical features

A total of 38 severe and critically ill children admitted to the PICU were included in this study, of whom 26 (68%) were classified as critical cases. Among these patients, 20 (53%) were male. The ages of the patients ranged from 30 days to under 17 years, with a median age of 25.5 (IQR: 12.5, 79.75) months. Children aged < 3 years accounted for 61% of the cases. Among the study population, 15 (39%) had received at least one dose of an inactivated COVID-19 vaccine (provided by Sinopharm CO.Ltd in Beijing or Wuhan, China and Kexing Biotch CO.Ltd in Shenzhen, China). Additionally, nine (24%) patients had pre-existing medical conditions. A total of 18 (47%) patients received IMV. Five of 38 patients (13%) developed cardiopulmonary arrest post-procedure, and four of five cases (80%) were successfully resuscitated.

Baseline characteristics and complications of the study population are summarized in **Table 1**. Several categorical variables, including fever, dyspnea, respiratory failure, laryngeal obstruction, ARDS, and the number of complications, were significantly associated with IMV. Among continuous variables, the PCIS, the PELOD-2, and the chest CT score (CO-RADS) were all significantly higher in the IMV group.

**Table 1:** Baseline information of 38 children with severe or critical COVID-19.

Item	Total (N=38)	Non-IMV (n=20)	IMV (n=18)	*p*- value
**Male n (%)**	20 (53)	9 (45)	11 (61)	0.352
**Age Group n (%)**				0.757
<1 y	9 (24)	6 (30)	3 (17)	0.454
1–2 yr	14 (37)	6 (30)	8 (44)	0.503
3–6 yr	7 (18)	4 (20)	3 (17)	1.0
7–17 yr	8 (21)	4 (20)	4 (22)	1.0
**Age (months)** median (Q1,Q3)	25.5 (12.5,79.75)	25 (5,85.25)	26 (15,84)	0.988
Disease severity classification **n (%)**				**0.000**
Severe disease	12 (32)	12 (60)	0	
Critical disease	26 (68)	8 (40)	18 (100)	
**Vaccine n (%)**	15 (39)	8 (40)	7 (39)	0.604
**Pre-existing medical conditions n (%)**	9 (24)	4 (15)	5 (22)	0.709
BPD	3 (8)	2 (10)	1 (6)	
Congenital atrial septal defect	1 (3)	–	1 (6)	**-**
Methylmalonic acidemia	1 (3)	–	1 (6)	**-**
Aplastic anemia	1 (3)	–	1 (6)	**-**
Diabetes	1 (3)	1 (5)	–	**-**
Epilepsy	1 (3)	1 (5)	–	**-**
Congenital hypothyroidism	1 (3)	–	1 (6)	**-**
**Symptoms n (%)**				
Fever	24 (63)	9 (45)	15 (83)	**0.02**
Cough	26 (68)	15 (75)	11 (61)	0.489
Dyspnea	27 (71)	9 (45)	18 (100)	**0.000**
Altered consciousness	26 (68)	15 (75)	11 (61)	0.489
Convulsion/seizures	15 (39)	10 (50)	5 (28)	0.198
Convulsive status	3 (8)	2 (10)	1 (6)	1.0
Nausea/Vomiting	6 (16)	3 (15)	3 (17)	1.0
Diarrhea	5 (13)	1 (5)	4 (22)	0.17
**Complications n (%)**				
Respiratory failure	25 (66)	7 (35)	18 (100)	**0.000**
Central nervous system	21 (55)	12 (60)	9 (50)	0.745
Myocardial damage	9 (24)	2 (10)	7 (39)	0.058
Laryngeal obstruction	6 (16)	0	6 (33)	**0.007**
DIC	3 (8)	0	3 (17)	0.097
ARDS	4 (11)	0	4 (22)	**0.041**
Number of complications	3 (2,5)	2 (1,4.5)	5 (3,7.5)	**0.001**
**Coinfection n (%)**	15 (39)	5 (25)	10 (56)	0.096
*Mycoplasma pneumoniae*	11 (29)	4 (20)	7 (39)	0.288
EB virus	2 (5)	0	2 (11)	0.218
Herpes simplex virus	2 (5)	1 (5)	1 (6)	1.0
PCIS median (Q1,Q3)	92 (88,98)	96 (92,100)	90 (81,93)	**0.001**
PELOD2 median (Q1,Q3)	1.5 (0,3)	0 (0,1)	2 (2,8.25)	**0.000**
P/F median (Q1,Q3)	268 (158,380)	456 (224,520)	260 (154,310)	0.057

Data are presented as the median (interquartile range) or number of patients (percentage) unless otherwise indicated. BPD, Bronchopulmonary dysplasia; PCIS, Pediatric Clinical Illness Score; PELOD2, pediatric logistic organ dysfunction-2 score.

*P*-values < .05 are in bold type.

P/F, PaO_2_/FiO_2_.

**Figure 1:**
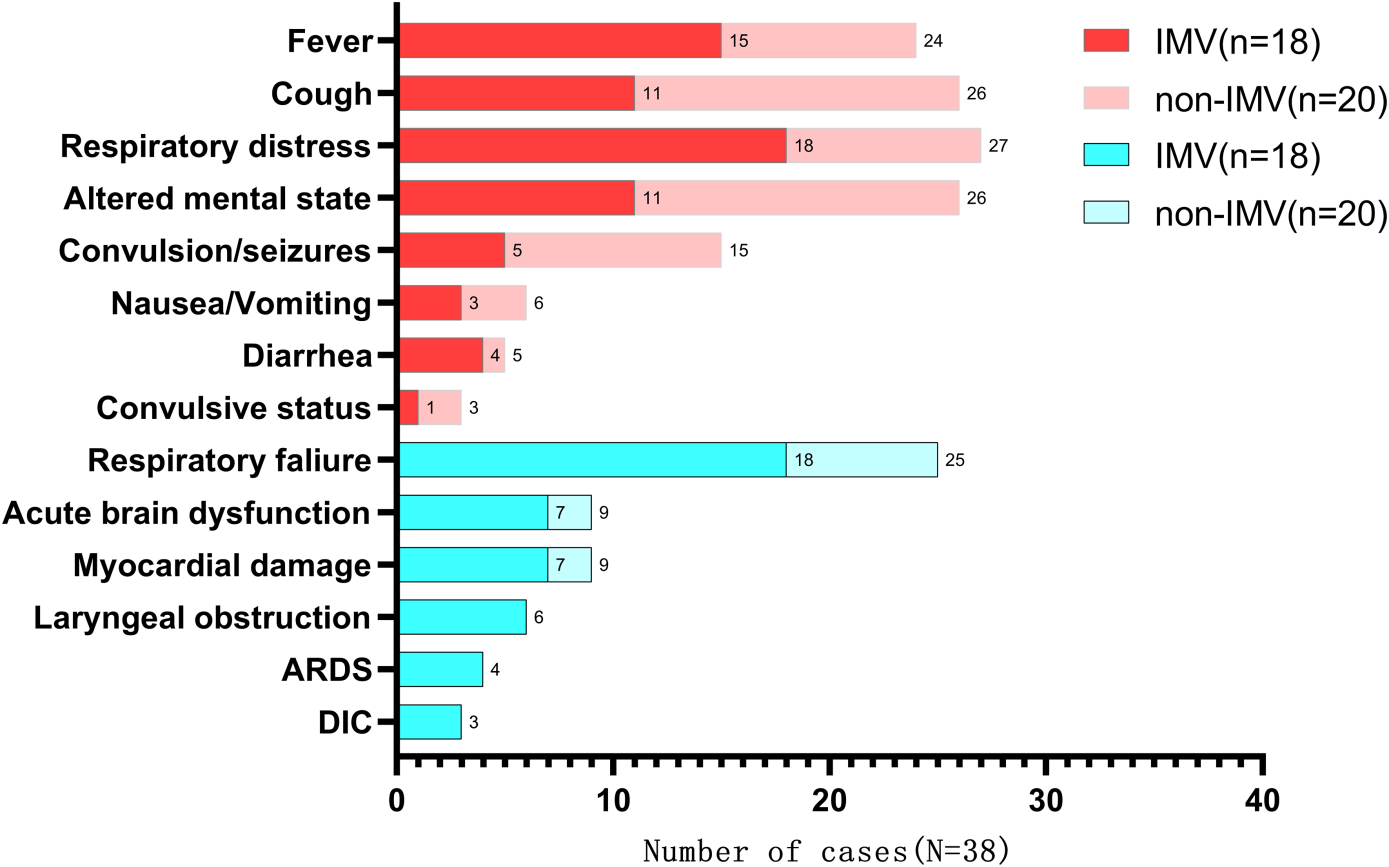
Main clinical manifestations and complications of severe or critically ill children with Omicron infection between IMV group and non-IMV group. The red part indicates clinical manifestations and the blue part indicates complications. CNS, central nervous system; DIC, disseminated intravascular coagulation.

**Figure 1** illustrates the clinical manifestations and complications in both IMV and non-IMV groups. The most common symptom was respiratory distress, followed by cough, altered mental state, and fever. The most significant complication was respiratory failure, followed by acute brain dysfunction.

### 3.2 Biochemical and radiological findings

Several continuous variables derived from laboratory data and the chest CT score based on CO-RADS were associated with the requirement for IMV support. Patients in the IMV group had lower mean platelet volume (MPV) levels and higher levels of creatine kinase (CK) and CK-MB isoenzyme. Additional laboratory findings are detailed in **Table 2**.

**Table 2:** Comparison of the laboratory indices between non-IMV and IMV groups.

Item	Total (N=38)	Non-IMV (n=20)	IMV (n=18)	*p*-value
Blood routine test Median (Q1,Q3)				
WBC counts cell (*10^9^/L)	6.2 (5.1,8.43)	6.045 (5.15,8.8)	6.3 (4.99,8.13)	0.953
Neutrophil counts cell (*10^9^/L)	3.55 (1.9,5.63)	3.5 (1.5,4.6)	3.95 (2.375,6.275)	0.365
Lymphocyte counts cell (*10^9^/L)	1.35 (0.9,3.23)	1.4 (0.925,3.475)	1.3 (0.85,3.225)	0.539
Hemoglobin levels (mg/L)	117 (101.5,128.5)	116.6 (99.25,127.25)	118 (105.5,129)	0.831
Platelet counts cell (*10^9^/L)	179.5 (127,276.5)	174.5 (138,257)	185.5 (101.25,386.25)	0.569
Eosinopenia counts cell (*10^9^/L)	0.15 (0,0.6)	0.35 (0,0.9)	0.1 (0,0.525)	0.367
MPV fL	8.45 (7.875,9.6)	9.1 (8.025,9.8)	8.25 (7.475,8.875)	**0.035**
NLR	2.29 (0.71,5.91)	1.61 (0.55,4.88)	3.194 (0.958,9.202)	0.219
PLR	123.32 (73.96,218.33)	88.35 (53.95,207.77)	149.09 (93.807,225.865)	0.169
SII	349.82 (131.68,1168.08)	247.31 (83.76,737.68)	559.15 (248,1370.88)	0.108
Cardiac biomarkers Median (Q1,Q3)				
CK (U/L)	188 (90.75,801.75)	118 (75.25,240.75)	294.5 (129.5,1117.75)	**0.044**
CKMB (U/L)	38.5 (19.68,51.25)	41.5 (20.5,47.75)	33 (16.6,90)	0.953
Troponin T (ng/L)	0.0155 (0.005,0.1183)	0.013 (0.00425,0.0835)	0.0165 (0.008,0.182)	0.497
Troponin I (ug/L)	0.0274 (0.0046,0.1340)	0.0061 (0.0039,0.1746)	0.0355 (0.0088,0.2088)	0.317
pro-BNP (pg/mL)	749.45 (223.25,1995.5)	545 (317.25,7502.75)	925.45 (182.5,1501)	0.671
CK - MB isoenzyme (ug/L)	5.3 (1.55,12.65)	2.6 (1.3,5.9)	9.45 (2.175,27.375)	**0.045**
LDH (U/L)	382.5 (316.25,837.25)	401 (308,1215.75)	365.5 (308.25,2034.25)	0.792
Liver function markers Median (Q1,Q3)				
ALT (U/L)	31.5 (17,81.5)	34 (17.5,73.25)	26.5 (17,121)	0.93
AST (U/L)	52.5 (42.75,174)	49 (39.75,174)	62.5 (43,215.5)	0.511
Albumin (g/L)	38.1 (34.3,41.65)	37.4 (34.2,41.5)	38.95 (33.825,43.2)	0.331
Kidney function Median (Q1,Q3)				
Cr (µmol/L)	24.7 (19.9,40.35)	23.45 (18.6,43.35)	27 (21.35,39.9)	0.604
Inflammation markers Median (Q1,Q3)				
CRP (mg/L)	6.6 (1.19,21)	6.6 (1.1775,17.4)	7.2 (1.55,24.98)	0.538
IL-6 (pg/mL)	17.15 (6.69,47.76)	16.17 (7.59,29.95)	22.99 (3.88,245.93)	0.233
PCT (ng/mL)	0.495 (0.195,2.35)	0.408 (0.231,1.56)	0.834 (0.15,2.905)	0.692
Coagulation function Median (Q1,Q3)				
PT (s)	12.8 (11.1,14.6)	12.2 (10.65,14.2)	13.3 (11.25,15.1)	0.409
APTT (s)	34 (30,43)	34.9 (32,43)	34 (28,45)	0.741
INR	1.2 (1.0,1.3)	1.1 (0.95,1.25)	1.25 (1.0,1.425)	0.157
Fib (g/L)	2.1 (1.5,2.6)	2 (1.455,2.515)	2.3 (1.475,2.725)	0.457
D-dimer (DDU)	314 (211,1242)	287 (208,1173)	376 (220,2180)	0.428
**Myoglobin (ug/L)** Median (Q1,Q3)	34.65 (16.58,150.68)	17.25 (14.48,36.35)	78.65 (22.5,364.38)	**0.012**
**Lactate (mmol/L)** Median (Q1,Q3)	1.7 (1.1,2.4)	1.3 (1.0,1.72)	2.05 (1.1,3.65)	0.111
**LAR**	0.0414 (0.0295,0.0701)	0.0390 (0.0265,0.0574)	0.0582 (0.0309,0.0809)	0.181
**FAR**	0.0550 (0.0369,0.0661)	0.0550 (0.0402,0.0655)	0.0562 (0.0367,0.0783)	0.717
**CAR**	0.1570 (0.0315,0.6274)	0.1570 (0.0295,0.5673)	0.1921 (0.0412,0.6860)	0.649
**LDH/Alb**	9.6062 (8.1433,24.9026)	9.7608 (8.2740,36.4935)	9.4517 (6.6339,15.1263)	0.622
**Glu (mmol/L)** Median (Q1,Q3)	5.6 (4.74,7.05)	5.32 (4.31,6.62)	6.2 (4.95,8.65)	0.223
Immunoglobulin (g/l) Median (Q1,Q3)				
IgA	0.4285 (0.1525,1.1465)	0.466 (0.08,1.25)	0.296 (0.154,1.183)	0.692
IgM	0.7935 (0.4953,1.1375)	0.787 (0.489,1.17)	0.8 (0.473,1.165)	0.925
IgG	6.35 (4.2875,9.0425)	7.33 (4.97,14.9)	5.23 (3.475,8.19)	0.168
T-lymphocyte subsets cell (/ul) Median (Q1,Q3)				
T-lymphocyte count	656 (510,1822.5)	868 (464.5,2159)	645 (528,1133)	0.63
Th count	313 (219,830)	435 (212.5,1570.5)	435 (212.5,1570)	0.324
Ts count	266 (177.5,518.5)	266 (154,590.5)	249.5 (192,465.75)	0.809
Natural killer cells count	185.5 (87,273)	213 (107,389.5)	145 (67,243)	0.222
B-lymphocyte count	480 (287,785.5)	712 (208.5,1381)	479 (360,526)	0.475
Ts/Th	1.21 (0.945,1.615)	1.3 (0.955,2.29)	1.1 (0.708,1.48)	0.293
**Chest CT Score**	2.5 (1,4.25)	1 (1,3)	4 (1.75,5)	**0.008**

Data are presented as the median (interquartile range) or number of patients (percentage) unless otherwise indicated. ALT, Alanine aminotransferase; AST, Aspartate aminotransferase; BNP, Brain natriuretic peptide; IL-6, Interleukin-6; CRP, C-reactive protein; LDH, Lactate dehydrogenase; LAR, Lactate/Albumin; FAR, Fibrinogen/Albumin; CAR, CRP/Albumin; NLR, neutrophil-lymphocyte ratio; PLR, Platelet-Lymphocyte Ratio; SII, systemic immune inflammation index; MLR, monocyte-lymphocyte ratio.

*P*-values < .05 are in bold type.

**Figure 2:**
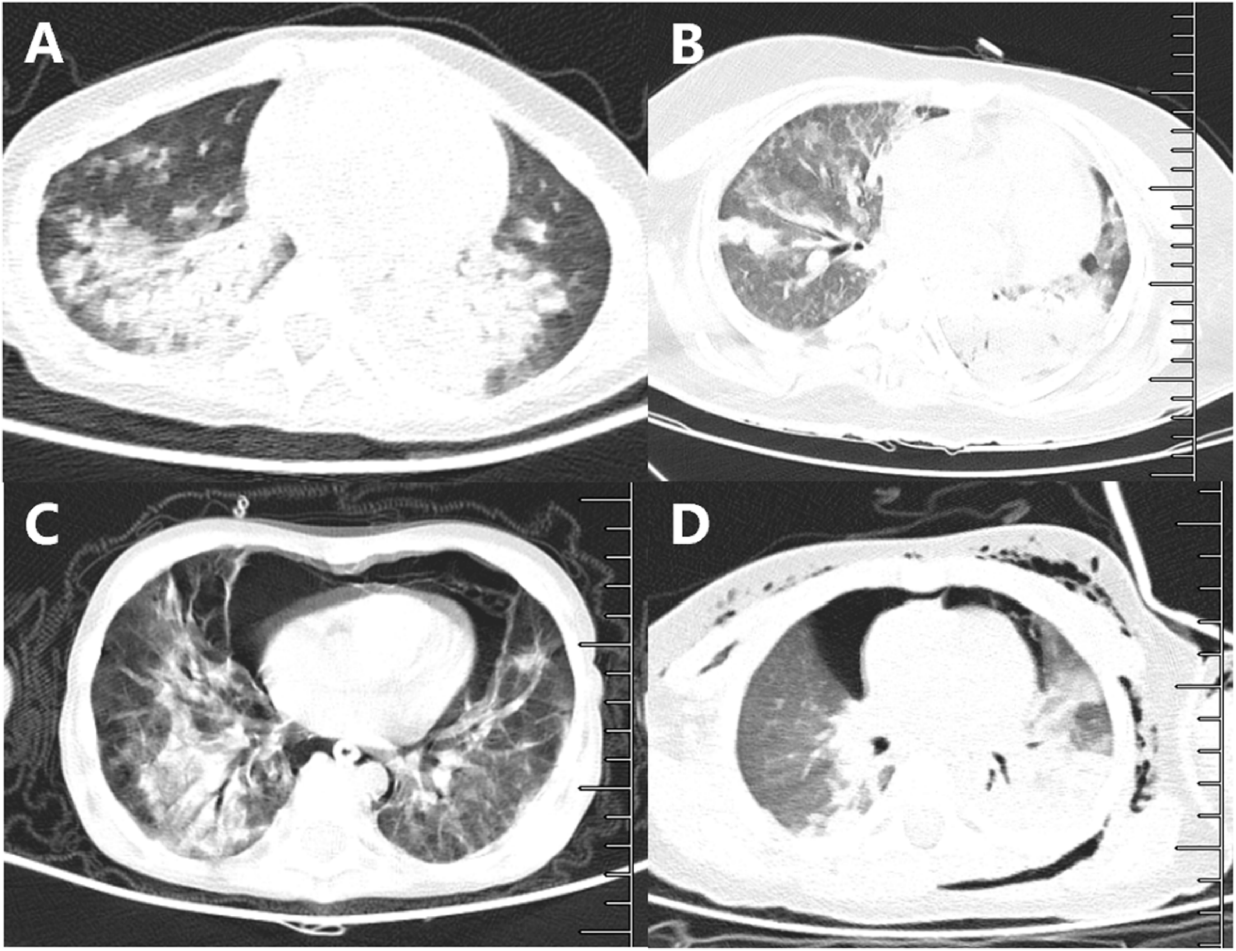
Chest CT findings of acute respiratory distress syndrome children with Omicron variant infection. Chest CT images of 4 critically ill children with ARDS: **(A)** a 4-year-old girl: ground-glass opacity and large areas of inflammation with partial consolidation; **(B)** a 16-year-old boy with methylmalonic acidemia; **(C)** a 6-year-old girl with mediastinal emphysema; **(D)** a 2-year-old boy with pneumothorax: partial effusion, who was coinfected with EBV.

Chest CT was performed in 34 (89%) patients, all of whom exhibited inflammatory lesions, with 27 (79%) showing bilateral lung involvement. Consolidation was present in 12 (35%) cases (**Supplementary Table 2**). The chest CT scans of four children with ARDS with Omicron infections are shown in **Figure 2**. As depicted in the figure, the image revealed ground-glass opacity and large inflammation areas with partial consolidation, with two cases showing pneumothorax. Additional chest CT scans are provided in **Supplementary Figure 1**.

**Figure 3:**
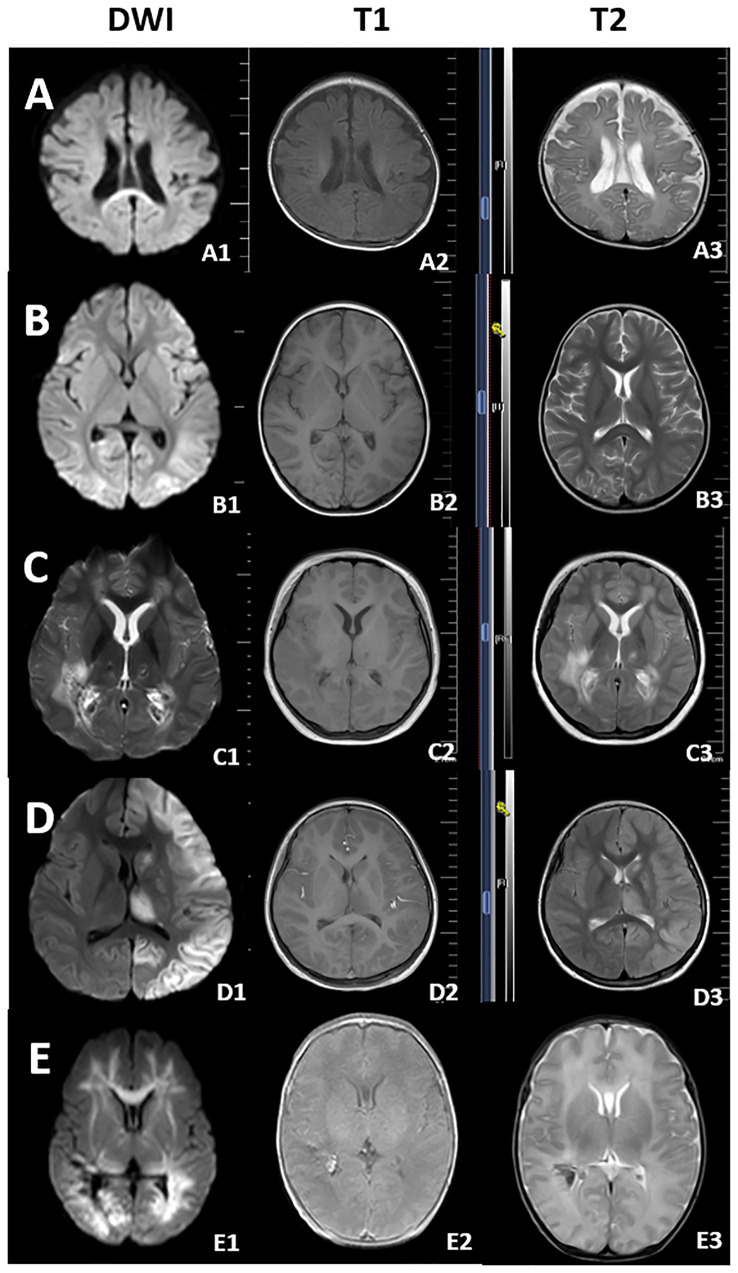
HEAD MRI of critically ill children infected with Omicron variant. **(A)** A 2-month-old boy with febrile seizures (For lung CT, see **Supplementary Figures D1, D2**). Cytotoxic edema was seen in the splenium of the corpus callosum. **(B)** A 28-month-old girl with febrile seizures. She had no obvious symptoms of respiratory system. Multiple cytotoxic edema lesions was seen in the brain. **(C)** A 11-year-old girl with febrile seizures. **(D)** A 9-year-old girl with status convulsion. She underwent shock and tracheal intubation and was discharged on the 8th day. **(E)** A 1-month-old boy, was admitted to the PICU at 39 weeks PMA. Head MRI shows extensive lesions in the brain.

Eighteen of 21 children (86%) with COVID-19-linked neurological disorders received head MRI, of whom 12 (67%) showed abnormalities. The most frequent clinical manifestations in these patients were unconsciousness and convulsions. MRI findings indicated that multiple brain regions were affected, either focally or diffusely. **Figure 3** displays typical MRI findings from five critically ill children with neurological disorders. Nine of the 12 (75%) patients with MRI abnormalities had bilateral brain involvement, with the most commonly affected regions being the paraventricular white matter and the frontal and occipital lobes. These abnormalities were associated with higher mRS scores at discharge. Detailed descriptions of MRI findings are available in **Supplementary Table 3**. Additionally, 17 (81%) patients received lumbar puncture for cerebrospinal fluid (CSF) analysis, but none tested positive for the new coronavirus in the CSF. Details are described in **Supplementary Table 2**.

### 3.3 Therapeutic and outcome data

Details on medications, respiratory support, and advanced therapies administered to patients, along with their outcomes, are summarized in **Table 3**. Children in the IMV group had significantly longer PICU stays (*p* < 0.001). In this group, two patients (5%) died: one patient succumbed to acute necrotizing encephalopathy, while the other died due to ARDS, lung air leak, and cutaneous emphysema. Meanwhile, eight patients had mRS scores ≥ 3 at discharge, including the two deceased patients. At 90-day follow-up, mRS scores ≥ 3 persisted in two patients.

**Table 3:** Treatment and outcomes of IMV and non-IMV groups with Omicron variant infection.

Item	Total (N=38)	Non-IMV (n=20)	IMV (n=18)	*p*-value
Treatment n (%)				
Antibiotics	34 (89)	16 (80)	18 (100)	0.107
Corticosteroids	31 (82)	13 (65)	18 (100)	**0.009**
Dexamethasone	16 (42)	4 (20)	12 (67)	**0.008**
Methylprednisolone	17 (45)	9 (45)	8 (44)	1.0
IVIG	15 (39)	7 (35)	8 (44)	0.741
Mannitol	13 (34)	7 (35)	6 (33)	1.0
Albumin	7 (18)	1 (5)	6 (33)	**0.038**
Antiviral drugs	3 (8)	0	3 (17)	0.097
Azvudine	1 (3)	0	1 (6)	-
Remdesivir	1 (3)	0	1 (6)	-
Monoclonal antibodies	1 (3)	0	1 (6)	-
Alprostadil	10 (26)	4 (20)	6 (33)	0.468
Adrenaline	3 (8)	0	3 (17)	-
Breathing support n (%)				
IMV≥96 h	16 (42)	0	16 (89)	**0.000**
IMV<96 h	2 (5)	0	2 (11)	-
Duration of IMV (h)	103.5 (0,170)	0	103.5 (0,170)	**<0.001**
High frequency ventilation	1 (3)	0	1 (6)	0.474
Inhaled nitric oxide therapy	2 (5)	0	2 (11)	0.218
Non-invasive assisted ventilation	11 (29)	5 (25)	6 (33)	0.724
Nasal cannula oxygen therapy	7 (18)	3 (15)	4 (22)	0.687
Advanced therapies n (%)				
Plasma exchange	7 (18)	4 (20)	3 (17)	1.0
CRRT	1 (3)	0	1 (6)	–
Tracheotomy	1 (3)	0	1 (6)	–
**LOS (days)** Median (Q1,Q3)	11.5 (7,18.25)	7.5 (5,11.75)	17 (12.25,22.5)	**0.000**
Outcomes n (%)				
LOS < 14 days and discharge	22 (58)	17 (85)	5 (28)	**0.001**
LOS ≥ 14 days and discharge	14 (37)	3 (15)	11 (61)	**0.006**
Death	2 (5)	0	2 (11)	0.218
**mRS score at discharge** Median (Q1,Q3)	0 (0.1.25)	0 (0,0)	0 (0,4)	0.298

Data are presented as the median (interquartile range) or number of patients (percentage). IVIG, Intravenous immunoglobulin; IMV, Invasive Mechanical Ventilation; CRRT, Continuous Renal Replacement Therapy; LOS, Length of hospital stays; IQR, interquartile range; mRS score, Modified Rankin Scale.

*P*-values < .05 are in bold type.

### 3.4 Analysis of factors associated with poor outcomes

Based on univariate analysis, sex and age were excluded from the subsequent multivariate analysis. In the multivariable model, the results showed that the chest CT score was the only independent predictor of IMV requirement in severe and critically ill children (OR 2.781 [95% CI 1.021–7.571]) (**Table 4**).

**Table 4:** Predictors of invasive mechanical ventilation.

Item	Non-IMV (n=20)	IMV (n=18)	OR (95%CI)	*p*-value
Univariable logistic regression analyses				
Disease severity classification (Critical)	8 (40)	18 (100)	–	0.998
PCIS ≤ 80	1 (5)	4 (22)	**-**	0.149
Number of complications≥3	8 (40)	16 (89)	12 (2.147-67.067)	**0.005**
Chest CT Score based on CO-RADS	1 (1,3)	4 (1.75,5)	2.028 (1.135,3.624)	**0.017**
CK≥188 (U/L)	4 (20)	9 (50)	4.0 (0.954,16.769)	0.058
CK-MB isoenzyme (ug/L) ≥10	1 (5)	9 (50)	14 (1.507,130.099)	**0.02**
Myoglobin (ug/L)≥34.65	1 (5)	6 (33.3)	6.75 (0.662-68.779)	0.107
IL-6≥20 pg/mL	4 (20)	10 (56)	5 (1.188-21.039)	**0.028**
Multivariable logistic regression analysis				
Number of complications≥3	8 (40)	16 (89)	4.919 (0.307-78.74)	0.26
CK-MB isoenzyme (ug/L) ≥10	1 (5)	9 (50)	**-**	0.999
Chest CT Score based on CO-RADS	1 (1,3)	4 (1.75,5)	2.781 (1.021-7.571)	**0.045**
IL-6≥20 pg/mL	4 (20)	10 (56)	4.045 (0.212-77.011)	0.353

Data are presented as the median (interquartile range) or number of patients (percentage). *P*-values < .05 are in bold type.

For secondary outcomes, multivariate analysis identified PELOD-2 (OR 2.717 [95% CI 1.011–7.299]) and serum AST levels (OR 14.766 [95% CI 1.395–156.308]) at admission as predictors of poor outcomes (death or prolonged PICU stay) in all severe and critically ill children with SARS-CoV-2 Omicron infection (**Table 5**).

**Table 5:** Predictors of death or PICU stay ≥ 14 days.

Variables	Univariable		Multivariable	
Univariable logistic regression analyses	OR (95%CI)	*p*-value	OR (95%CI)	*p*-value
Number of complications≥3	21.667 (2.412,194.649)	**0.006**	–	–
**mRS score**	2.01 (1.173,3.445)	**0.011**	**-**	–
**PELOD2≥3**	23.8 (3.99,141.963)	**0.001**	2.717 (1.011,7.299)	**0.047**
**Neutrophil counts cell ≥3.55 × 10^9^/L**	4.714 (1.178,18.861)	**0.028**	–	**-**
**CK≥188 (U/L)**	4.714 (1.178,18.861)	**0.028**	–	**-**
**Troponin I (ug/L) ≥0.0274**	4.4 (1.041,18.599)	**0.044**	–	–
**AST≥52.5 (U/L)**	11.556 (2.411,55.392)	**0.002**	14.766 (1.395,156.308)	**0.025**
**IL-6≥20 pg/mL**	8.25 (1.65,41.247)	**0.008**	–	–
**PCT (ng/mL)≥0.5**	6.5 (1.467,28.804)	**0.014**	–	**-**
**PT≥12.8 (s)**	12.133 (2.405,61.202)	**0.003**	–	–
**INR≥1.2**	12.133 (2.405,61.202)	**0.003**	–	–
**D-dimer (DDU)≥314**	12.133 (2.405,61.202)	**0.003**	4.51 (0.494,41.169)	0.182
**Lactate≥1.7 (mmol/l)**	5.28 (1.196,23.317)	**0.028**	–	–

*P*-values < .05 are in bold type.

## 4 Discussion

In this study, we described the clinical characteristics of severe and critically ill children diagnosed with the first wave of SARS-CoV-2 Omicron infection who were admitted to the PICU of the National Regional Children’s Medical Center in Northeast China. Our findings indicate that chest CT score based on CO-RADS, serum AST levels at admission, and the PELOD-2 score may predict adverse outcomes in this specific population.

Our data showed that 61% of severely and critically ill children were younger than 3 years old. A statistical analysis of children in France with acute COVID-19 infections in the PICU found that 45.1% of patients were aged <2 years (excluding those under 1 month), while 60.7% were aged <5 years (Recher et al., 2023). Similarly, a British study reported that 61% (36/59) of PICU patients were aged <5 years (Ward et al., 2023). With the emergence of the Omicron variant, mathematical modeling and quantitative analyses of empirical data predict that first-time infections may occur at younger ages due to rising population immunity from prior Omicron infections and vaccination efforts (Koelle et al., 2022). A plausible explanation for this trend is that children aged <3 years remain unvaccinated (Tachikawa et al., 2025), making them more vulnerable to severe initial infections and increasing likelihood of requiring PICU admission.

Comorbidities have been consistently associated with severe SARS-CoV-2 infection in pediatric populations, with studies reporting that 15.6% to 83% of pediatric patients have at least one pre-existing condition across various cohorts (Ungar et al., 2023; Kulkarni et al., 2024). Our study aligns with this range, revealing that 24% of patients had comorbidities, with 13% requiring IMV and 8% receiving non-IMV. Although the clinical symptoms of Omicron infection are mild in the vast majority of healthy children, those with comorbidities may experience further deterioration of underlying conditions and a decline in quality of life (Dryden et al., 2022; Jassat et al., 2023; Calcaterra et al., 2024). Compared to the Alpha variant, the Omicron group had a higher prevalence of comorbidities, worse initial laboratory data, and higher in-hospital mortality rates (40.6% vs 15.2%, *p* = 0.004). The Charlson Comorbidity Index was identified as an independent risk factor for in-hospital mortality (Cheng et al., 2025). These findings highlight the heightened risk of adverse outcomes in pediatric patients with comorbidities, emphasizing the need for closer monitoring and targeted interventions.

At admission, 25 children (66%) were diagnosed with respiratory failure, and four (16%) developed ARDS. A previous study in southern China (Guangdong) supports our findings (Lin et al., 2024b). Similarly, research on critically ill adults and children in the northeastern United States (New York) found that 90% of patients who developed ARDS initially presented with dyspnea (Derespina et al., 2020). Accurate identification of these presentations, combined with the early administration of respiratory support, may improve patient outcomes by minimizing disease progression and alleviating stress in healthcare facilities (Mizuno et al., 2023). Initial studies during the pandemic linked respiratory system impairment at hospital admission to adverse outcomes with ARDS, serving as an independent predictor of disease severity (Ni et al., 2024). Some research suggests that COVID-19 comorbidities increase with latitude, but we found no evidence to confirm an association between geographic location and complications or comorbidities in children.

COVID-19 is known to cause neurological complications (Bhalala et al., 2022). Studies report that approximately one-third of hospitalized children with Omicron infection experience neurological disorders, such as convulsions (Choi et al., 2023; Tang L. et al., 2024), with higher incidence rates in severe cases (LaRovere et al., 2023). A study by Lin et al. found that 70% of critically ill children with SARS-CoV-2 Omicron infection (Lin et al., 2024b) developed encephalopathy. Among the cohort, 21 (55%) had neurological complications, with convulsion being the most common manifestation (39%). However, we could not determine whether SARS-CoV-2 infection was the primary cause of these neurological symptoms. There are some potential pathogenic mechanisms of SARS-CoV-2 infection affecting the central nervous system (CNS): direct invasion of the CNS, excessive release of pro-inflammatory cytokines, and the immune escape effect of the virus (Viviani et al., 2003; Hautala et al., 2021; Kurd et al., 2021; Shi et al., 2023).

In our study, 12 patients had abnormal head MRI findings. A single-center study conducted in the United States found that the most prevalent imaging findings among adults were nonspecific white matter microangiopathy (55.4%), chronic infarcts (19.4%), acute or subacute ischemic infarcts (5.4%), and acute hemorrhage (4.5%) (Radmanesh et al., 2020). Additionally, a study focusing on ICU patients in France reported that 23% (6/26) of patients experienced cerebrovascular events (Cleret de Langavant et al., 2021). Research in South China involving children supports our findings, indicating that all cases exhibited bilateral involvement, particularly in the thalamus and basal ganglia (Lin et al., 2024a). Possible reasons for these differences in brain involvement or clinical manifestations may relate to age and cardiovascular risk factors (Lu et al., 2024), or the likelihood of children developing acute necrotizing encephalopathy may be higher (Karami et al., 2023). Multicenter cohort studies on the adverse neurological functional outcomes and exploring factors are needed.

Our research shows that chest CT score is associated with the need for IMV in severe and critically ill children. Li et al. did not calculate chest CT scores but described CT findings of consolidation, linear opacities, crazy-paving pattern, bronchial wall thickening, high CT scores, and extrapulmonary lesions as features of severe or critical COVID-19 pneumonia (Li et al., 2020). A study conducted in Brazil on non-invasive respiratory support and IMV concluded that alternating non-invasive respiratory support with HFNO increased IMV rates, regardless of comorbidities and chest CT scores among patients with COVID-19. The chest CT scores were significantly different among all groups (da Cruz et al., 2024). For immunocompromised patients, the total chest CT score was associated with longer hospitalization and ICU admission (Ghadery et al., 2024). Compared with prior studies, the results in our cohort accurately predicted the need for IMV in severe and critically ill children with first-time Omicron infection. Our findings further support the predictive value of chest CT scores for IMV requirements, which could be enhanced by combining CT analysis with other clinical parameters (Orlandi et al., 2021). The results above suggest that the chest CT score based on CO-RADS could be an important factor in predicting poor outcomes for severe and critically ill children.

The study showed that PELOD-2 and serum AST levels ≥52.5 U/L at admission were associated with increased mortality or prolonged PICU stays. A multi-center survey on prolonged mechanical ventilation (MV) showed that the use of vasoactive agents and higher PELOD-2 scores at the time of the prolonged MV diagnosis were significantly associated with an increased risk of prolonged MV-related death. Meanwhile, early rehabilitation intervention was identified as crucial for improving patient outcomes (Zhang et al., 2024). Several studies used the PELOD-2 to predict mortality and prognosis (El-Nawawy et al., 2017; Schlapbach et al., 2018; Lee et al., 2022). These findings suggest that the predictive power of PELOD-2 may vary across specific subpopulations.

The results of this study indicated that abnormalities in various laboratory indicators were more pronounced in critically ill children within the IMV group. Notably, serum AST levels were a predictor of PICU duration. Other indicators included CK-MB isoenzyme (µg/L) ≥10, IL-6 ≥20 pg/mL, and coagulation indices (PT, INR, D-dimer). Early Omicron infection may induce subclinical cholangiocyte damage through a multifactorial and complex pathogenic process, differing from earlier strains of SARS-CoV-2 (Iheanacho and Enechukwu, 2022). Cao et al. (2024) observed that patients with abnormal liver enzyme levels exhibited significantly elevated inflammatory markers, including PCT, IL-6, and CRP. IL-6, as an inflammatory factor, may be attributed to stress responses in the liver, characterized by the release of major acute-phase cytokines in response to Omicron infection (Dufour et al., 2022). A study on adenovirus (ADV) infection found that AST levels were also associated with longer hospital stays in children (Tang S. et al., 2024). Based on these findings, serum AST levels can be considered a reliable indicator of prolonged PICU stays.

This study has some limitations. First, the relatively small sample size might limit the generalizability of our conclusions to patients in other regions. Second, since this was a retrospective study, potential biases and confounding factors might have influenced the results. Additionally, long-term follow-up data were not collected. Finally, standardized scores were not used to assess disease severity in patients, which should be considered in future research.

## 5 Conclusions

Respiratory failure and COVID-19-associated neurological disorders are major complications in severe and critically ill children with first-wave SARS-CoV-2 Omicron infection. Chest CT score based on CO-RADS may predict severe and critically ill children with SARS-CoV-2 Omicron infection requiring intensive mechanical ventilation. Additionally, serum AST levels at admission and the PELOD-2 score may predict prolonged PICU stays and mortality, helping to avert the development of potentially life-threatening complications.

## Data Availability

The raw data supporting the conclusions of this article will be made available by the authors, without undue reservation.
