# Supplementary material for: Characteristics and outcomes in severe and critically ill children with first wave SARS-CoV-2 Omicron infection in Northeast China
Source: Front Cell Infect Microbiol. 2025 Apr 15;15:1495783. doi: 10.3389/fcimb.2025.1495783 (PMC12037530; doi:10.3389/fcimb.2025.1495783)
Supplement: Supplementary file 1 [file Presentation1.pdf]

Supplementary Material

Supplementary Figure and Tables

Supplementary Figure

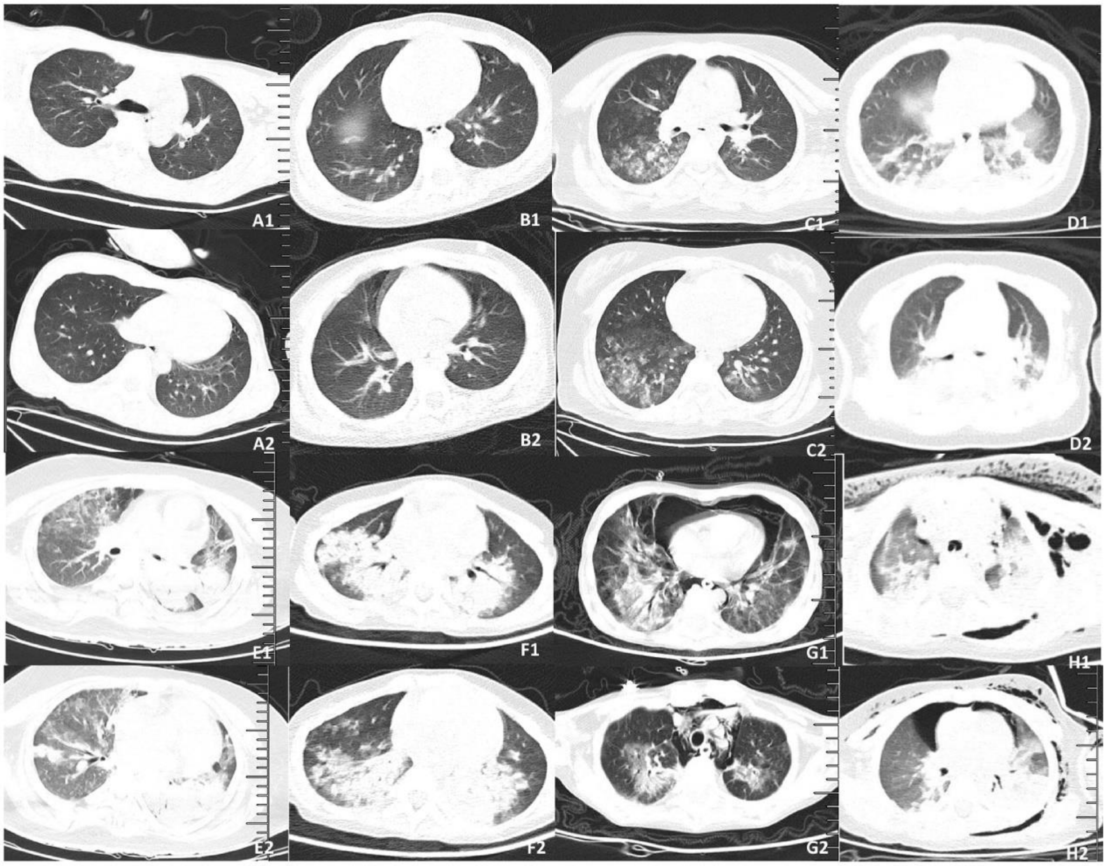

Supplementary Figure 1. Chest CT findings of children with Omicron variant infection

(A)A 13-year-old boy, admitted with seizures and unconsciousness. Lung CT showed bilateral inflammation. (B)A 15-month-old girl, admitted with dyspnea and fever. Lung CT showed bilateral inflammation. (C)An 11-year-old girl, admitted with vomiting and febrile seizures. Lung CT shows multiple patch shadow and nodular shadows. She was discharged on the 20th hospital day, general condition was good. (D)A 2-month-old boy, extubated. He was in deep coma with central respiratory failure on admission. He was discharged on the 19th hospital day, general condition was good. (E)A 16-year-old boy with methylmalonic acidemia, was in a light coma and had frequent convulsions on admission. Lung CT showed bilateral multi-lobar inflammation with consolidations. (F)A 4-year-old girl with high fever (40.1℃) and a blood oxygen saturation of 76% without supplemental oxygen on admission. Lung CT shows a wide range of inflammatory lesions. (G) A 6-year-old girl with aplastic anemia (post-transplantation). Lung CT showed bilateral multi-lobar inflammation, pneumothorax, and mediastinal emphysema. Unfortunately, she eventually died of respiratory failure on the 30th hospital day. (H)A 23-month-old boy with dyspnea, was transferred to our hospital with endotracheal intubation, loss of consciousness on admission. He was diagnosed with severe pneumonia and pneumothorax.

Supplemental Table 1. Differences in the COVID-19 severity classification protocol for clinical management between WHO and NHCC

	WHO	NHCC's guidelines
**Severity classification**		
**Mild:**	Meeting the case definition for COVID-19 without evidence of viral pneumonia or hypoxia	The main manifestations are symptoms of upper respiratory tract infection: dry throat, sore throat, cough, fever and so on
**Moderate:**	**Adolescent** or adult with clinical signs of pneumonia (fever, cough, dyspnea , fast breathing) but no signs of severe pneumonia, including SpO2 ≥ 90% on room air.<br><br>**Child** with cough or difficulty breathing + fast breathing and/or chest indrawing and no signs of severe pneumonia .[a]	Meeting all of the following criteria:<br><br>(1)Persistent high fever> 3 days, or (and) symptoms such as cough and shortness of breath,<br><br>(2) RR(Respiratory rate) <30 bpm and SpO2 >93% on room air at rest.<br><br>(3)Characteristic imaging manifestations of COVID-19 pneumonia were observed
**Severe**:	Severe pneumonia<br><br>**Adolescent or adult** with clinical signs of pneumonia (fever, cough, dyspnea) plus one of the following: respiratory rate > 30 breaths/min, severe respiratory distress, or SpO2 < 90% on room air.<br><br>**Child:** with clinical signs of pneumonia (cough or difficulty breathing + fast breathing or chest wall indrawing) + at least one of the following:<br><br>SpO2 < 90%<br><br>Very severe chest indrawing, grunting, central cyanosis, or presence of any other general danger sign (inability to breastfeed or drink, lethargy or unconsciousness or convulsions).<br><br>The diagnosis can be made on clinical grounds; chest imaging (radiograph, CT, ultrasound) may assist in diagnosis and help identify or exclude pulmonary complications	**Adolescent or adult:** meet any of the following criteria and can't be explained by reasons other than COVID-19 infection:<br><br>(1)Shortness of breath, RR ≥30/min;<br><br>(2)SpO2≤ 93% on room air;<br><br>(3)PaO2/FiO2≤300 mmHg[b] ;<br><br>(4)Progressively worsening clinical symptoms, with chest imaging showing the lesions significant progression of lesions by more than 50% within 24-48 hours.<br><br>**Child**: meet any of the following criteria:<br><br>(1)Persistent high fever> 3 days;<br><br>(2)Shortness of breath ,[c]<br><br>(3)SpO2 ≤93% on room air at rest;<br><br>(4)Alar flapping, three concave signs or wheezing;<br><br>(5)Unconsciousness, convulsions;<br><br>(6)Difficulty feeding with signs of dehydration.
**Critical:**	With acute respiratory distress syndrome (ARDS), sepsis, septic shock, acute thrombosis, or MIS-C	Meeting any of the following criteria:

	(1)Respiratory failure requiring mechanical ventilation,;<br><br>(2)Shock,;<br><br>(3)Admission to the intensive care unit (ICU) for other organ dysfunction/

a.Fast breathing: < 2 months:⩾60 breaths/min; 2 – 11 months:⩾50; 1 – 5 years:⩾40. b. Should be corrected at high altitudes. c.Shortness of breath: < 2 months:⩾60 breaths/min; 2 – 12 months:⩾50; 1 – 5 years:⩾40；>5 years:⩾30（Excluding the influences of fever, crying, and other symptoms）

Supplementary Table 2. Results of imaging and other laboratory examinations in children with Omicron variant infection

Items	Non-IMV group (n=20)	IMV group (n=18)
Chest CT(n=34)	16	18
Reduced permeability	16	17
Bilateral inflammatory lesions	13	14
Unilateral inflammatory lesions	2	3
Solid	1	11
Larynx CT(n=4)	0	4
Abnormal	-	4
Video Electroencephalography (n=17)	9	8
Abnormal	5	7
Cerebrospinal fluid (n=17)	8	9
Elevated CSF pressure	2	3
Abnormal cell count	2	1
Abnormal glucose	0	0
Abnormal protein	2	2
Cytokine panel examination (n =12)	7	5
IL-2 (pg/mL) Median (Q1,Q3)	4.57(3.038,6.66)	3.135(2.15,4.773)

IL-4 (pg/mL)	Median (Q1,Q3)	9.095(5.278,10.803)	5.74(2.11,8.558)
IL-10 (pg/mL)	Median (Q1,Q3)	1.785(1.048,3.338)	5.74(1.553,22.655)
IL-17 (pg/mL)	Median (Q1,Q3)	6.035(4.33,8.903)	4.74(2.485,9.003)
INF-γ (pg/mL)	Median (Q1,Q3)	12.04(8.018,16.815)	9.165(6.268,22.613)
TNF-α (pg/mL)	Median (Q1,Q3)	4.745(2.358,5.923)	4.33(2.308,7.715)
IL-1β (pg/mL)	Median (Q1,Q3)	2.51(2.48,4.738)	4.325(2.368,19.135)
IL-12p70 (pg/mL)	Median (Q1,Q3)	4.45(3.04,7.933)	3.135(2.15,47.753)

Data are the median (interquartile range) or number of patients (percentage).

Abbreviations: CSF, Cerebrospinal fluid; INF-γ, Interferon-γ; TNF-α, Tumor necrosis factor-α; IL, Interleukin.

Supplementary Table 3. Sites and manifestations of abnormal MRI in 12 patients

Sites of involvement	Bilateral cytotoxic edema	Left cytotoxic edema	Right cytotoxic edema	Hemorrhage	Other
Paraventricular leukomalacia	4	1	0	0	2
Frontal lobe	5	1	0	0	0
Occipital lobe	4	0	0	1(right)	0
Basal ganglia	3	1	0	0	0
Temporal lobe	3	0	0	1(right)	0
Parietal lobe	3	0	0	1(right)	0

Cerebellar	2	0	0	0	0
Hypothalamus	2	0	0	0	0
Callose	2	0	0	0	0
Brainstem	1	0	0	0	0
